# 

**Published:** 1899-09

**Authors:** 


SUBSCRIPTION $1 00.

PUBLISHED MONTHLY.

### ATLANTA JOURNAL-RECORD OF MEDICINE.

Successor to Atlanta Medical and Surgical Journal, Established 1855, and Southern Medical Record, Established 1870.

Vol. I. Atlanta, Ga., September, 1899. No. 6.

